# Primary thoracic muscle tuberculosis: two case reports

**DOI:** 10.1186/s13256-016-0996-2

**Published:** 2016-08-16

**Authors:** Leonidas Grigorakos, Vassilis Sgountzos, Daria Lazarescu, Sophia Simopoulou, Magda Gkouni, Nikolaos Markou, Vassilis Tamvakis

**Affiliations:** 1 Faculty of Nursing, National and Kapodistrian University of Athens, Athens, Greece; 2 Intensive Care, General Trauma and Accident Hospital of Athens KAT, Athens, Greece; 3 Sotiria Chest Diseases Hospital, Athens, Greece; 4 Peripheral Anticancer Oncology Hospital of Athens “Agios Savvas”, Athens, Greece; 5 Intensive Care at Latseion Burn Center, Thriasio General Hospital, Elefsina, Greece

**Keywords:** Muscle tuberculosis, Skeletal muscle, Extrapulmonary tuberculosis, Case report

## Abstract

**Background:**

The aim of this case report is to present our experience with two very rare cases of thoracic muscle tuberculosis. Muscle tuberculosis, as a primary disease, can only be detected in cases in which mycobacteria have been transplanted to a muscle through an infected needle.

**Case presentations:**

Case 1 is a 38-year-old immigrant man and Case 2 is a 24-year-old immigrant man, both originating from Sub-Saharan African Countries; they presented in the past two years to our hospital with swellings at the base of the hemithorax and were diagnosed as having muscle tuberculosis. Administration of anti-tuberculosis chemotherapy caused: (a) diminution of inflammation, (b) diminution of the size of local fusiform injury, and (c) clinical improvement.

**Conclusions:**

Thoracic muscle tuberculosis should be considered to be one of the etiologies of muscular disease in European countries with a high incidence of immigrants originating from endemic geographical areas.

## Background

Tuberculosis (TB) is considered a “re-emerging disease” due to its increasing incidence in the twenty-first century. Extrapulmonary involvement constitutes about 20 % of all cases of TB in immunocompetent patients of which about one-tenth involve the musculoskeletal system (mostly spondylitis, osteomyelitis, or arthritis) [1].

Involvement of skeletal muscle without coexisting active disease is very rare, with the incidence of primary muscle TB being reported as 0.015 % [2]. Primary disease can only be detected in cases in which mycobacteria have been transplanted to a muscle through an infected needle during a thoracentesis or an intramuscular injection. Usually, patients with muscle TB first manifest symptoms of TB (same as in other cases of extrapulmonary TB); muscle TB results from the hematogenous transmission of mycobacteria from a latent pulmonary or nodal focus, and not an obvious one. Thus, cases of muscle TB have been described as occurring through the latent hematogenous dissemination of TB [3, 4].

Muscle tissue is resistant to mycobacteria TB infestation. The reason for the resistance has yet to be clarified. It has been attributed to poor oxygen in muscle tissue, the scarcity of reticulo-epithelial tissue, and the presence of lactic acid within skeletal muscles [5].

In the first study that described muscle TB, only four such cases were found after performing an autopsy in 2244 patients with TB [6]. Skeletal muscles can be infected with muscle TB if a neighboring tuberculous focus (that is, in bones through the rupture and erosion of the walls of an active peripheral tuberculous cavity) spreads (that is, from cold paravertebral abscess to the abdominal muscles) [7] (Grigorakos L, Sgountzos V, Simopoulou S, Lazarescu D, Mavropanou D, Georgiadou A, Tamvakis V: Psoas muscle tuberculosis in Greece: Report of two cases, forthcoming). 

Muscle TB is frequently misdiagnosed as sarcoma, soft tissue tumor, parasitic infectious hydatid cyst, fungal infection, hematoma, or lipoma [8]. These potential misdiagnoses led us to describe our experience with two such cases.

## Case presentations

We present Case 1, a 38-year-old immigrant man, and Case 2, a 24-year-old immigrant man, both originating from Sub-Saharan African Countries, who presented in the past two years to our hospital with pain at the base of their hemithorax (Table 1). Both patients were submitted to detailed examination. A laboratory examination, a sputum examination for acid-fast bacilli (five samples) and chest X-rays did not show pathological findings. Their tuberculin skin tests were positive with the induration measuring 22 mm and 25 mm, respectively. An ophthalmological examination and thorax computed tomography (CT) revealed tubercles in the retina of both eyes as well as tumors at the base of hemithorax.

**Table 1:** Patients’ characteristics

Patients’ characteristics	Case 1	Case 2
Age (years)	38	24
Sex	Male	Male
Weight (kg)	62	58
Height (m)	1.71	1.70
Symptoms at admission	Swelling and pain at the base of right hemithorax	Swelling and pain at the base of left hemithorax
Tuberculin skin test	22 mm	25 mm

Histological samples taken from the base of the hemithorax of Case 1 and Case 2 (two broad samples of maximal diameter 2.5 cm and 2 cm respectively, whitish-brown color and tough-elastic composition) revealed that these included a part of striated muscle displaying an increase in the intramuscular and perimuscular connective substratum, and an inflammatory infiltration, mostly by lymphocytes. In some regions, tuberculomas of epithelioid cells and some Langhans giant cells were observed.

Fine-needle aspiration (FNA) of the thoracic tumor revealed positive for acid-fast bacilli by Ziehl–Neelsen (ZN) stain while the culture in Löwenstein–Jensen medium (LJ medium) was also positive to muscle TB. Molecular methods of sample examination (GenoType MTBDRplus) revealed muscle TB without mutations compatible with resistance to isoniazid (INH) and rifampicin (RMP).

After Case 1 and Case 2 were diagnosed as having muscle TB, they were subjected to anti-TB therapy in our clinic, receiving: (a) daily dosages of 300 mg INH and 600 mg RMP administered orally, (b) daily 2.5×500 mg of ethambutol (EMB) in the form of Dexambutol tablets and 20mg/kg PO qDay pyrazinamide (PZA), and (c) daily dosage of 25 mg B6 administered orally.

Within 20 days, swellings at the base of the hemithorax of both Case 1 and Case 2 decreased and the size of their fusiform sores was greatly reduced; they receded completely over time. Both patients felt better and their appetite has improved. Case 1 and Case 2 were discharged in good health and they continue their treatment; they attend bimonthly check-ups at the anti-TB department of our hospital.

## Discussion

We have described two cases of muscle TB of the hemithorax at the height of the middle line towards the outer intercostal muscles of the abdomen, which are rare in the newer international literature [4, 9]. The location was due to a latent hematogenous spread of *Mycobacterium tuberculosis* from some unknown (latent) pulmonary or nodal focus, which is not always found in pulmonary X-rays or in a necropsy. The consequent infection of the thoracic muscle via the connecting tissue was not possible in our case due to lack of local thoracentesis.

About half of the cases of musculoskeletal TB have evidence of active or healed pulmonary TB. A single site of involvement is generally seen, but multiple locations are not uncommon [10].

The lack of findings of caseous necrosis in a biopsy does not exclude muscle TB. Thus, findings of CT scans of the involved muscle are helpful in differential diagnosis as they demonstrate findings suggestive of muscle TB (mediastinal lymphadenopathy with low central density in patients with chest wall muscle TB; the absence of adjacent venous thrombosis or cellulitis which usually occurs in patients with pyogenic myositis) [11].

Ultimately, a definite diagnosis needs to be based on histologic and microbiologic studies in order to avoid misdiagnosis. In our case, from the very beginning we excluded the possibility of sarcoidosis and confirmed thoracic muscle TB diagnosis through: (a) a clearly positive tuberculin skin test; (b) the negative biochemical and other laboratory tests (blood calcium, angiotensin-converting enzyme, and so on); and (c) the results of radiological tests (bilateral hilar lymph enlargement). Eventually, diagnosis was well established by the rather favorable effect of the combined antituberculous chemotherapy within a rather short time span.

## Conclusions

Although rare, thoracic muscle TB is an existing clinical entity and should be considered to be one of the etiologies of muscular disease, not only in areas where TB is endemic but also in European countries with a high incidence of immigrants or refugees originating from such areas. As it may pose a diagnostic challenge, we underline the importance of keeping high suspicion of muscle TB in patients with hematogenous dissemination. The definite diagnosis should pursue a clinically directed multidisciplinary approach based on microbiologic and histologic examination.

## Abbreviations

CT, computed tomography; EMB, ethambutol; FNA, fine-needle aspiration; INH, isoniazid; LJ medium, Löwenstein–Jensen medium; PZA, pyrazinamide; RMP, rifampicin; TB, tuberculosis; ZN, Ziehl–Neelsen
